# Establishment and Maintenance of Repressed Chromatin States on Dosage-Compensated Sex Chromosomes

**DOI:** 10.3390/biom16030386

**Published:** 2026-03-04

**Authors:** Joshua Eduful, Lily LeSarge, Györgyi Csankovszki

**Affiliations:** Department of Molecular, Cellular and Developmental Biology, University of Michigan, Ann Arbor, MI 48109, USA; jeduful@umich.edu (J.E.);

**Keywords:** dosage compensation, epigenetics, condensin, maintenance, XIST

## Abstract

Sex chromosome imbalance is a genetic challenge in species with unequal X-chromosome numbers. Organisms have developed distinct strategies to control this imbalance through a process called dosage compensation. These strategies include X-chromosome inactivation in mammals mediated by the XIST long noncoding RNA and proteins recruited by XIST, and X-linked hypertranscription in male *Drosophila* driven by the Male-Specific Lethal (MSL) complex. In *Caenorhabditis elegans*, gene expression is downregulated from each of the two X chromosomes of hermaphrodites by half, thereby matching the levels in XO males. This is mediated by a specialized condensin-containing protein complex, the Dosage Compensation Complex (DCC). In all cases, the chromatin states on the sex chromosomes must be first established and then maintained for the entire lifetime of the organism. Although mammals and nematodes both use repression to achieve dosage compensation, the mechanisms are very different. Here, we summarize recent advances on how repressive chromatin states are established and maintained, with a focus on contrasting *C. elegans* dosage compensation to XIST-mediated X-chromosome inactivation. We review how specialized chromosome topology, repressive chromatin modifications, and higher-order nuclear architecture are established and maintained to achieve sex-specific regulation of the X chromosomes and highlight key outstanding questions and future research directions.

## 1. Introduction

In organisms that use chromosome-based sex determination, the number and composition of sex chromosomes play a key role in sex determination. For instance, in mammals, males have XY and females have XX chromosomes, and sex is determined by the presence or absence of the Y-linked *SRY* gene [1]. In *Drosophila melanogaster,* males have one X-chromosome and a Y chromosome, and females have two X chromosomes. In *C. elegans,* males have one X (and no Y), and hermaphrodites have two X chromosomes. In *Drosophila* and *C. elegans,* sex is determined by the ratio of X chromosomes to autosomes [2]. The difference in the number of X chromosomes between sexes of organisms creates a chromosomal imbalance between the two sexes. Without compensating for the difference in the number of X chromosomes, it results in potentially deleterious differences in gene dosage and disruption in cellular homeostasis and development. For example, in *C. elegans*, the differences in X-chromosome number results in X-linked gene expression which is two-fold higher in hermaphrodites compared to males. To solve this problem, organisms employ diverse mechanisms to control gene expression levels between their sexes in a process called dosage compensation. Dosage compensation is a fundamental biological process that ensures balanced expression of X-linked genes between individuals with different numbers of X chromosomes.

Different organisms use distinct mechanisms of dosage compensation. In 1961, Mary Lyon formulated the X chromosome inactivation hypothesis, proposing that one X chromosome in female mammals is transcriptionally silenced early in development, thereby equalizing X-linked gene expression between XX females and XY males [3]. Mammals therefore undergo dosage compensation by transcriptionally silencing one of the two X chromosomes in females [3,4]. Mammalian X-chromosome inactivation (XCI) is driven by the long noncoding RNA XIST expressed exclusively from the inactive X-chromosome [4,5,6,7]. Silencing is mediated by proteins recruited to the X, including SPEN, hnRNP-K, and Polycomb repressive complexes (PRC), and SmcHD1 [4]. XCI is tightly coupled to loss of pluripotency and cellular differentiation [8]. In pluripotent embryonic stem cells and early embryos, core pluripotency factors such as OCT4, NANOG, and SOX2 repress the Xist long noncoding RNA and maintain both X chromosomes in an active state; upon differentiation and exit from pluripotency, repression of Xist is relieved, leading to its upregulation and initiation of XCI [4,9,10,11,12]. Complete silencing of X-linked genes also requires differentiation-dependent mechanisms [8].

**Figure 1:**
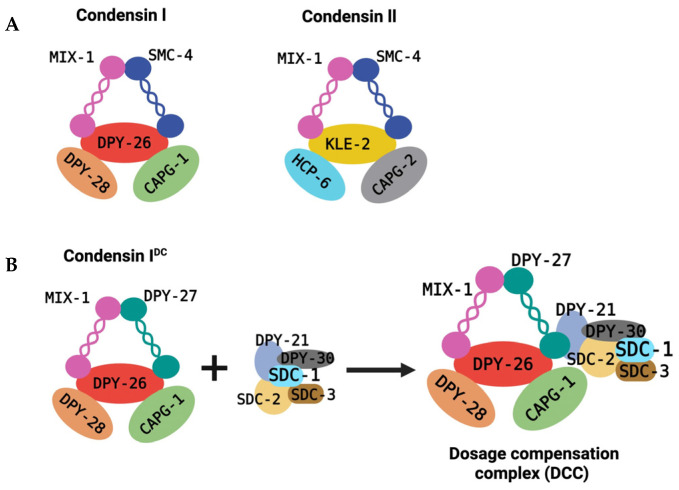
The *C. elegans* DCC contains a condensin-like complex. (**A**) Two canonical condensins present in *C. elegans* with mitotic functions. (**B**) Condensin I^DC^ differs from condensin I only by the presence of DPY-27 instead of SMC-4. Condensin I^DC^ associates with additional proteins to form the DCC.

In contrast, *Caenorhabditis elegans* uses a different dosage compensatory strategy in which XX hermaphrodites reduce transcription from each X chromosome by approximately half, thereby matching the gene expression output of XO males [13]. This is accomplished by a group of proteins referred to as Dosage Compensation Complex (DCC). The members of DCC were first identified through the discovery of sex-specific lethal mutations which result in hermaphrodite-only lethality and have no impact on males. It was observed that mutations in six genes including *sdc-2*, *sdc-3*, *dpy-26*, *dpy-27, dpy-28*, and *dpy-30* were associated with hermaphrodite-specific lethality [14,15,16]. Some of the DCC proteins form a complex related to condensin. Condensins are evolutionarily conserved protein complexes that associate with chromosomes and play key roles in chromosome compaction and segregation in mitosis and meiosis [17,18]. Higher eukaryotes have two main types of condensin: condensin I and condensin II [19,20,21] (Figure 1A), which play key but distinct roles in mitosis and meiosis [22]. Interestingly, *C. elegans* has a third condensin called condensin I^DC^ which plays an exclusive role in dosage compensation in *C. elegans* hermaphrodites [23] (Figure 1B). Condensin I and condensin I^DC^ share some common protein subunits, including the Structural Maintenance Chromosome 2 (SMC2) protein MIX-1, chromosome-associated polypeptide G (CAPG-1), DPY-26, and DPY-28 [23,24]. The difference between condensin I and condensin I^DC^ stems from the presence of DPY-27 in condensin I^DC^, whose paralog is SMC-4 in condensin I. The only subunit unique to condensin I^DC^, DPY-27, therefore, ensures the dosage compensation-specific function of condensin I^DC^ [23,25]. Condensin I^DC^, together with additional proteins, including SDC-1, -2, and -3, as well as DPY-30 and DPY-21, form the complete DCC [26] (Figure 1B). As in mammals, initiation of dosage compensation in *C. elegans* is linked to loss of pluripotency and differentiation [27].

Mammals and *Caenorhabditis elegans* are the best studied examples of chromosome-wide repressive mechanisms used to achieve sex chromosome dosage compensation. It is interesting that different evolutionary lineages adapted such fundamentally different underlying molecular mechanisms to achieve the same goal. While in female mammals, one X chromosome is shut down and the other stays active, in hermaphroditic *C. elegans,* the mechanism involves chromosome-wide transcriptional repression of both Xs without fully silencing the X chromosomes. From an epigenetic perspective, dosage compensation raises two central questions. First, how is a chromosome-wide regulatory state initially established in a developmentally controlled and sex-specific manner? Second, once established, how is this state maintained through successive rounds of DNA replication, mitosis, and cellular differentiation? Not only do mammals and *C. elegans* use widely different molecular machinery to establish repression on the target chromosome(s) (long non-coding RNA versus a condensin-like complex), the fundamental logic behind mechanisms that sustain dosage compensation over time are distinct as well. In mammals, the initiating machinery triggers additional mechanisms that render maintenance of X-inactivation largely independent of the initial trigger [4,9,28,29], and thus is a phenomenon truly “epigenetic” in nature. By contrast, in *C. elegans*, maintenance of dosage compensation continues to rely on its initiating machinery, the DCC, to maintain dosage compensation over time [30].

In this review, we synthesize recent advances in understanding how repressive chromatin states are established and maintained, with a particular focus on contrasting *Caenorhabditis elegans* dosage compensation with XIST-mediated X-chromosome inactivation in mammals. We examine how specialized chromosome topology, repressive chromatin modifications, and higher-order nuclear architecture cooperate to achieve stable, sex-specific regulation of X-linked gene expression, and we highlight key unresolved questions and important directions for future research.

## 2. Mechanism of Dosage Compensation Initiation and Establishment in Mammals

Mammals undergo dosage compensation through XCI, a process that transcriptionally silences one X chromosome in XX females. Mammalian dosage compensation relies on long noncoding RNA-mediated recruitment of chromatin regulators. Recent work has emphasized that chromatin modifications and higher-order chromosome architecture is not just a consequence of XCI but a central driver of both initiation and establishment of the inactive X chromosome [4,31].

### 2.1. Developmental Timing and Context of Mammalian Dosage Compensation

In mammals, dosage compensation through X chromosome inactivation (XCI) is tightly coupled to cellular state. In mouse embryos, an initial form of imprinted XCI occurs during early cleavage stages, but this silencing is transient and restricted to extraembryonic lineages [32,33]. Cells of the inner cell mass subsequently reactivate the inactive X-chromosome, returning to a state with two active X chromosomes [34,35,36]. Canonical, random XCI is then initiated after implantation, coincident with exit from pluripotency and onset of differentiation [4,37]. Indeed, studies have shown that complete and stable X-linked gene silencing in mammals requires differentiation [8].

Recent work has highlighted that mammalian dosage compensation is not a single, uniform process, but rather proceeds through distinct developmental stages. Human preimplantation embryos employ the mechanism of X-chromosome dampening, in which transcription from both X chromosomes is reduced without full inactivation of either chromosome [4,38,39,40,41]. This is similar to the dosage compensation mechanism used by *C. elegans*, suggesting that partial, reversible dampening may represent an evolutionarily conserved solution that mammals adopt briefly before committing to stable XCI. While both early human embryos and *C. elegans* use chromosome dampening, the molecular machineries behind these processes are different. At these early stages in humans, XIST RNA and additional non-coding RNA XACT are expressed from both X chromosomes [42]. Although not fully understood, dampening seems to require XIST and one of its interacting protein partners SPEN [41], similar to the machinery involved in XCI (see below), and different from the machinery used in *C. elegans.*

### 2.2. Genetic and Developmental Signals in Establishment of Mammalian Dosage Compensation

**Figure 2:**
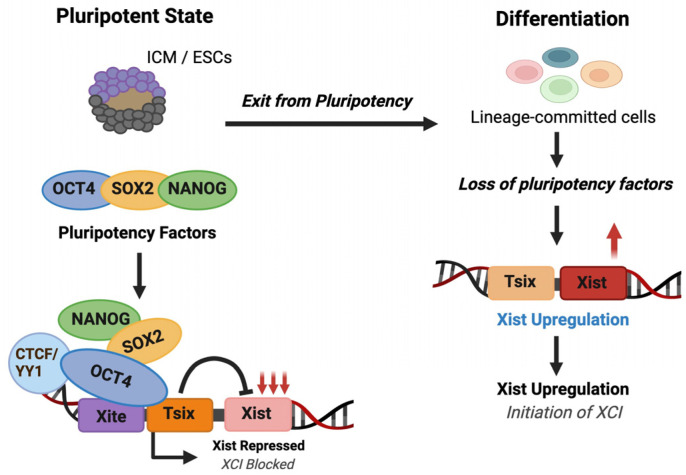
Pluripotency-dependent control of Xist expression during initiation of X-chromosome inactivation in mammals. In the early stages of embryogenesis, cells of the ICM or embryonic stem cells (ESCs) are pluripotent. Core transcription factors, OCT4, SOX2, and NANOG maintain pluripotency and repress Xist expression. XCI initiation is blocked until cells start to exit the pluripotent state. As cells differentiate, there is a decline in pluripotency factor activity; hence, repression of Xist is relieved. Xist is then activated to trigger the onset of XCI.

In mammals, initiation of XCI is controlled by developmental state, and this regulation occurs through a bona fide pluripotency network. Central to mammalian dosage compensation is the activation of the long noncoding RNA XIST, which is transcribed from the X-inactivation center and acts as the primary initiator of chromosome-wide silencing [5,6,7]. In mammals, regulation of XIST expression is closely tied to pluripotency and early differentiation. In the early stages of embryogenesis, cells of the inner cell mass (ICM) and epiblast exist in a pluripotent state, defined by the activity of core transcription factors such as OCT4, SOX2, and NANOG, which maintain pluripotency and prevent premature differentiation [43,44,45]. They also actively repress Xist expression in undifferentiated cells through multiple mechanisms. At this stage, pluripotency factors bind regulatory elements around the Xist locus and cooperate to suppress Xist transcription, thereby ensuring that XCI initiation does not occur while a cell is pluripotent [10,37,46,47]. One of the pluripotency factors which regulate XCI is OCT4. OCT4 interacts with chromatin architectural proteins such as CTCF and YY1 and binds to regulatory regions of the X-inactivation center, including Tsix and Xite, antagonizing Xist expression [10,47,48,49] (Figure 2). This binding enhances Tsix expression and facilitates X-chromosome pairing and counting, which is required for proper choice of the inactive X, while preventing inappropriate Xist upregulation in pluripotent cells [10]. Xist therefore remains repressed in pluripotent cells; hence, XCI initiation is blocked until cells start to exit the pluripotent state (Figure 2).

PRC1 and PRC2 control the onset and progression of cellular differentiation in mammals, acting as epigenetic “brakes” to silence developmental genes in pluripotent stem cells (PSCs) [50,51]. Through deposition of H3K27me3 at developmental gene loci, PRC2 helps silence pluripotency programs and create a chromatin environment permissive for Xist expression and XCI initiation. As cells differentiate and pluripotency networks collapse, repression of Xist is relieved, enabling Xist upregulation and initiation of XCI [12]. This promotes the upregulation of Xist by one of the X chromosomes, leading to the initiation of XCI (Figure 2). Thus, the decline in pluripotency factor activity constitutes a molecular permissive signal for Xist activation and the onset of X-chromosome silencing.

### 2.3. Establishment of X-Chromosome Silencing Through Xist-Dependent Protein Recruitment

Mammals use Xist RNA as the chromosome-recognition and recruitment platform. Upon activation, Xist is expressed from the inactive X chromosome and spreads in cis across the X chromosome from which they originate, remaining strictly bound to that chromosome [7,8]. This spreading is not guided by linear DNA sequence but by three-dimensional chromatin architecture. Xist initially associates with genomic regions that are spatially proximal to its transcription site, using existing nuclear organization as a scaffold for chromosome-wide coverage [4,52]. This process results in the formation of discrete Xist RNA granules within the X-chromosome territory.

**Figure 3:**
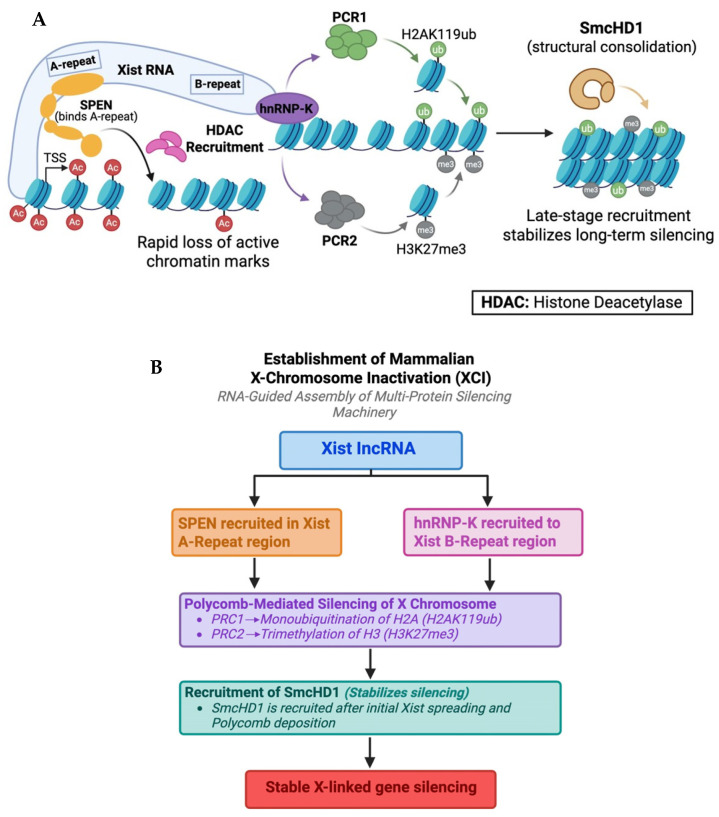
Mammalian XCI is established through RNA-guided assembly of multi-protein repressive machinery. (**A**) SPEN binds to the A-repeat region of Xist RNA. The B-repeat region of Xist binds hnRNP-K, which functions as a scaffold for recruitment of Polycomb repressive complexes PRC1 and PRC2. PRC1 catalyzes monoubiquitination of histone H2A at lysine 119 (H2AK119ub), while PRC2 deposits trimethylation of histone H3 at lysine 27 (H3K27me3). Later, SmcHD1 is recruited to consolidate gene silencing. (**B**) Summary of how XCI is established in mammals.

Mammalian XCI is established through RNA-guided assembly of a multi-protein repressive machinery. During this stage, SPEN, a transcriptional repressor, binds to the A-repeat region of Xist RNA (Figure 3A). SPEN is required for silencing most X-linked genes and acts predominantly by recruiting histone deacetylases, leading to rapid loss of active chromatin marks at promoters and gene bodies [53,54,55,56,57] and disruption of either the Xist A-repeat or SPEN results in a near-complete failure of transcriptional repression. Xist recruits additional factors that promote stable chromatin remodeling. The B-repeat region of Xist binds hnRNP-K, which functions as a scaffold for recruitment of Polycomb repressive complexes PRC1 and PRC2 [58,59,60,61,62,63]. PRC1 catalyzes monoubiquitination of histone H2A at lysine 119 (H2AK119ub), while PRC2 deposits trimethylation of histone H3 at lysine 27 (H3K27me3) (Figure 3A). These modifications spread across the X chromosome and reinforce transcriptional repression. An additional, less well-understood, chromatin mark, histone H4 lysine 20 monomethylation (H4K20me1) appears on the inactive X chromosome at about the same time [64,65].

As XCI progresses, other structural regulators are recruited to the inactive X chromosome. Among these, SmcHD1 helps in consolidating gene silencing, particularly at genes that are repressed later during differentiation [8,58,66] (Figure 3A). SmcHD1 recruitment occurs after initial Xist spreading and Polycomb deposition. The delayed recruitment of SmcHD1 demonstrates that the establishment of XCI is multistep, with distinct classes of genes silenced at different stages. Together, these findings reveal a model of the establishment of dosage compensation in mammals: Xist RNA first recruits transcriptional repressors to dampen gene expression, then recruits Polycomb complexes to impose chromatin-based repression, and finally recruits architectural proteins that stabilize the inactive state (Figure 3B).

### 2.4. Reorganization of X-Chromosome Architecture: Xist Granules, Protein Condensation, and Compartment Formation

Beyond recruitment of transcriptional repressors and chromatin modifiers, establishment of mammalian dosage compensation relies on a reorganization of three-dimensional chromosome architecture. XCI is associated with the collapse of active regulatory architecture and formation of a repressive nuclear compartment driven by Xist RNA. Xist does not coat chromatin uniformly. Instead, Xist accumulates in discrete RNA granules within the X chromosome territory, with approximately 50 granules per inactive X chromosome, each containing a small number of Xist transcripts [4,67,68]. Given the scale of the X-chromosome and the number of genes subject to silencing, this organization promotes the amplification of silencing signals beyond direct RNA–chromatin interactions.

This amplification occurs through protein condensation. Xist recruits a large cohort of interacting proteins via its conserved repeat elements. These proteins engage in protein–protein interactions, many of which are low-affinity but highly multivalent. As a result, Xist-associated factors form highly dynamic supramolecular assemblies, often referred to as supramolecular complexes (SMACs) [68]. These generate regions of high local protein density surrounding Xist granules, leading to the concentration of repressive and architectural regulators within the X-chromosome territory. Disruption of Xist-mediated condensation impairs transcriptional silencing, even when Xist RNA remains chromosome-associated. This demonstrates that compartment formation is not a secondary consequence of repression but a mechanistic requirement for it [68]. Within these condensed regions, transcriptional activators and components of the transcription machinery are depleted, largely due to reduced chromatin binding capacity. This creates a chromatin environment in which transcription is inhibited not only by epigenetic marks but also by physical exclusion of activating factors.

**Figure 4:**
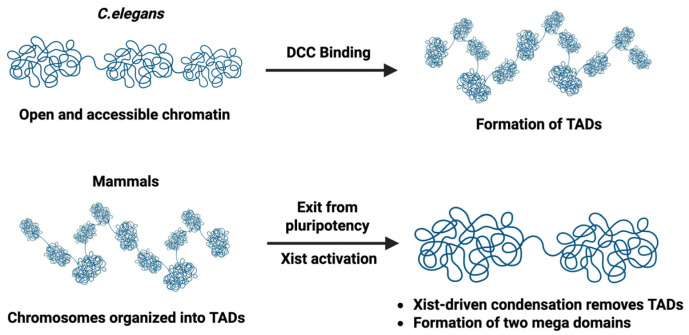
Different structural changes are induced on the X chromosomes in *C. elegans* and mammals. In *C. elegans*, the DCC establishes new TADs to modulate transcription while in mammals, the inactive X-chromosome loses conventional TADs instead, and adopts a simplified, compartmentalized state of two megadomains.

Following protein condensation formation, the large-scale chromatin architecture of the X chromosome is remodeled. The inactive X-chromosome loses conventional topologically associated domains (TADs) and long-range enhancer–promoter interactions, which are a characteristic of active chromosomes. Instead, the inactive X adopts a simplified, compartmentalized state of two megadomains with reduced regulatory complexity [69,70,71] (Figure 4). Disruption of this structure by deleting the Dxz4 locus at the megadomain border does not lead to widespread X-chromosome reactivation, suggesting that the structure may contribute to silencing, in a manner that is redundant with other silencing mechanisms, but it is not strictly required [69,72,73].

## 3. Initiation and Establishment of Dosage Compensation in *C. elegans*

### 3.1. Developmental Timing and Biological Context of Dosage Compensation Onset

Genetic and developmental signals which trigger the onset of dosage compensation in mammals and *C. elegans* are different, although in both cases, the onset of dosage compensation is linked to loss of plasticity and the onset of differentiation. In *C. elegans*, dosage compensation is initiated as cells lose their ability to adopt alternative fates [27]. This loss of developmental plasticity in *C. elegans* is regulated by Polycomb repressive complex 2 (PRC2) [74]. The PRC2 catalytic subunit MES-2, the worm homolog of mammalian EZH2, promotes the transition from a plastic embryonic state to differentiation by repressing genes associated with alternative developmental programs. In *mes-2* mutants, embryonic cells retain an extended capacity for fate reprogramming, demonstrating that PRC2 is required for timely exit from developmental plasticity [74]. In these mutants, dosage compensation onset is also delayed [27]. Thus, although *C. elegans* does not possess a pluripotent stem cell state analogous to mammalian embryonic stem cells [75,76], PRC2 nonetheless plays a conserved role in coordinating developmental progression toward differentiation.

Together, the onset of dosage is regulated by exit from a highly plastic developmental state in both mammals and in *C. elegans*. In *C. elegans*, this transition is regulated by PRC2-mediated restriction of developmental plasticity and permits DCC recruitment to the X chromosomes, whereas in mammals, dissolution of pluripotency networks enables Xist activation and initiation of XCI. Despite this similarity, the molecular mechanisms and regulatory outcomes remain distinct.

**Figure 5:**
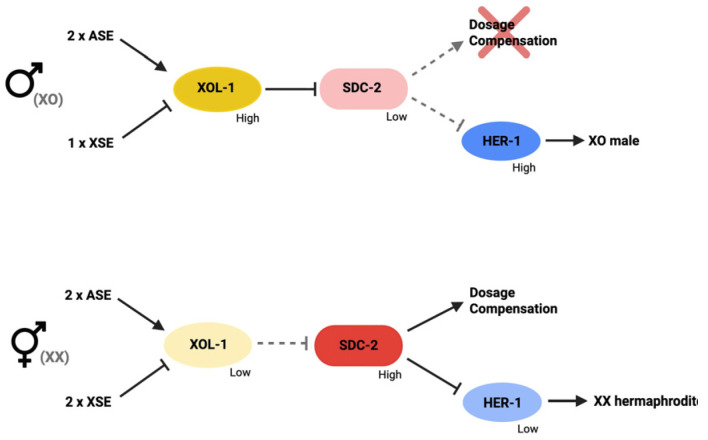
SDC-2 is regulated by XOL-1 to mediate both sex determination and dosage compensation. When the X:A ratio is 0.5, XOL-1 levels become high, and represses SDC-2. HER-1 expression then becomes high, and the embryo develops to become a male and dosage compensation is not initiated. When X:A ratio is 1, levels of XOL-1 are low and SDC-2 expression levels become high. SDC-2 triggers the onset of dosage compensation and blocks the expression of HER-1, and the embryo develops into a hermaphrodite.

The onset of dosage compensation marks the earliest phase at which organisms respond to sex chromosome imbalance by initiating chromosome-wide regulatory control. In *C. elegans*, initiation of dosage compensation occurs early in embryogenesis, with the recruitment of DCC onto X chromosome occurring around the 30–40 cell stage [77]. This timing ensures that dosage compensation is established before the onset of high transcriptional activity. One of the key proteins in the DCC is SDC-2. SDC-2 plays a critical role in both sex determination and dosage compensation. It is negatively regulated by a protein called XOL-1 in the sex determination pathway (Figure 5). XOL-1, XO lethal, is the master switch gene whose activity is determined by the X: A ratio [2,78,79,80,81]. Unlike in mammals, where sex determination and dosage compensation are regulated by distinct mechanisms, in *C. elegans,* these two processes are linked and regulated by the activity of XOL-1. When the X:A ratio is 0.5, XOL-1 levels become high, which then represses the expression of SDC-2. With SDC-2 expression being low, HER-1 expression then becomes high, and the embryo develops to become a male, and dosage compensation is not initiated. In *xol-1* mutant XO animals, SDC-2 is expressed, which leads to repression of the single male X and lethality [78]. On the other hand, when X:A ratio is 1, the expression levels of XOL-1 become low and SDC-2 expression levels become high. SDC-2 then triggers the onset of dosage compensation and blocks the expression of HER-1, and the embryo develops into a hermaphrodite (Figure 5). Other DCC members are maternally loaded into oocytes. SDC-2, however, is not maternally loaded and it is only produced in XX hermaphrodite embryos. This ensures that DCC assembly only occurs in XX embryos and reveals that SDC-2 is the key protein required for the assembly of DCC on X chromosome at the onset of dosage compensation.

### 3.2. Recruitment of DCC on the X Chromosome in XX Hermaphrodites

**Figure 6:**
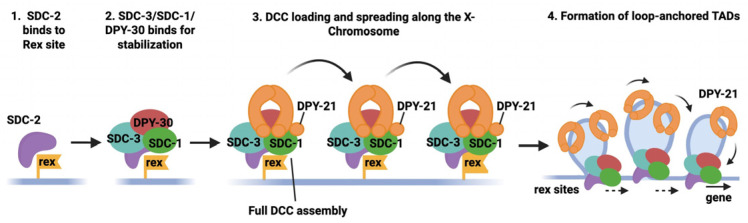
Hierarchical recruitment of DCC and establishment of dosage compensation. SDC-2 is the first DCC component to associate with the X chromosome by binding to rex sites, initiating DCC assembly. SDC-3 and DPY-30 are subsequently recruited in an SDC-2-dependent manner and stabilize early DCC binding. This enables loading of the condensin I^DC^ complex (shown in orange), followed by its spreading along the X chromosome. At later embryonic stages, DPY-21 associates with the DCC and contributes to higher-order chromosome organization.

DCC can distinguish between X chromosomes and autosomes. X chromosomes have distinct DNA motifs, called *rex* (recruitment element on X), which serve as an initial recruitment site for DCC binding. The rex site is a 12 bp consensus motif enriched on the X chromosome [82,83,84]. Chromatin immunoprecipitation analysis revealed that these sites correspond to the highest peaks of DCC binding on the X [84,85]. Recruitment of DCC to the X chromosomes occurs through a hierarchical and ordered assembly pathway that ensures sex-specific and chromosome-specific regulation (Figure 6). While in mammals, the lncRNA XIST plays a pivotal role in recruiting proteins to the Xs; no such RNA has been identified in *C. elegans.* Instead, the earliest limiting factor in this process is SDC-2, a hermaphrodite-specific protein whose expression is restricted to XX embryos, and which serves as the master initiator of dosage compensation [77]. SDC-2 is therefore the first DCC component to associate with the X-chromosome, although whether it can directly bind DNA is unknown. SDC-2 can bind to the X-chromosome even in the absence of other DCC members [77,86]. This further affirms that SDC-2 is the key protein which first binds to the X chromosomes and triggers DCC assembly.

Following SDC-2 binding to the *rex* sites, additional regulatory proteins are recruited in an SDC-2-dependent manner. SDC-3 and DPY-30 are among the earliest factors to associate with the X after SDC-2 and function to stabilize DCC assembly and facilitate recruitment of downstream components. These proteins do not independently recognize the X chromosome, instead, their localization requires prior SDC-2 binding, placing them downstream in the recruitment hierarchy [77,86]. SDC-3 is a zinc-finger protein which assists SDC-2 in the recruitment of other DCC members on the X chromosomes. DPY-30 contributes to the stabilization of early DCC assembly and recruitment to the X.

Once the SDC proteins are in place, the condensin I^DC^ complex is recruited to the X chromosome. This complex includes the dosage-compensation-specific SMC subunit DPY-27, its shared SMC partner MIX-1, and the non-SMC subunits DPY-26, DPY-28, and CAPG-1. Condensin I^DC^ loading depends on the prior action of SDC-2 and SDC-3 and cannot occur independently of these factors [23,26]. After initial loading at *rex* sites, condensin I^DC^ spreads along the length of the X chromosome, occupying both recruitment sites and transcriptionally active regions, thereby enabling chromosome-wide repression of X-linked gene expression [84,85,86]. DPY-21 later localizes to the DCC on the X at around the bean-to-comma stage (200–500 cells) [83]. Together, these observations support a model in which DCC binds to the X chromosome proceeds in a strictly ordered sequence, beginning with SDC-2, followed by stabilization through SDC-3 and DPY-30, the loading and spreading of condensin I^D^, and culminating in the binding of DPY-21.

### 3.3. Establishment of Dosage-Compensated State in C. elegans

Dosage compensation initiation is characterized by the binding of the DCC to the X chromosome. After this, dosage compensation is established through changes in chromosome topology, histone modification, chromatin accessibility, transcriptional activity and nuclear organization. Establishment of dosage compensation begins immediately after DCC recruitment to the X and continues through embryogenesis. In this subsection, we have discussed major events which lead to the establishment of dosage compensation.

#### 3.3.1. Formation of a Condensin I ^DC^ Structural Scaffold

DCC recruitment is followed by the assembly of a condensin-like structural scaffold along the X chromosomes. Following recruitment to rex sites, condensin I^DC^ spreads along the X chromosomes to occupy a broader set of secondary sites that lack autonomous recruitment activity. DCC activity in hermaphrodites leads to X-chromosome compaction in interphase nuclei [87]. At higher resolution, Hi-C analysis demonstrated that recruitment of the DCC induces large-scale remodeling of X-chromosome topology in XX hermaphrodites [88,89]. In contrast to autosomes, the compensated X chromosomes adopt a distinctive three-dimensional organization characterized by regularly spaced self-interacting domains with strengthened boundary insulation. These self-interacting domains resemble topologically associated domains (TAD) in mammals. Many of these domain boundaries coincide with high-affinity rex sites, indicating that DCC recruitment not only targets the X-chromosome but also defines its topological architecture [88,90,91,92].

The involvement of condensin in *C. elegans* dosage compensation raises the question of whether condensin plays similar roles in other organisms. Although condensin complexes are broadly conserved as drivers of loop extrusion and chromosome compaction, the activity of condensin I^DC^ in dosage compensation is specific. Rather than inducing uniform compaction as in mitosis, condensin I^DC^ establishes a stable interphase architecture composed of insulated domains and altered long-range interaction frequencies [88,91]. Mammalian condensin I is cytoplasmic during interphase, but condensin II is nuclear [20,21], suggesting that condensin II may be able to contribute to shaping interphase chromosome architecture, similar to *C. elegans* condensin I^DC^. However, condensin II activity is inhibited in interphase by the binding of MCPH1, and at the onset of mitosis, a phosphorylation-dependent switch triggers the displacement of MCPH1, the binding of M18BP1, and condensin’s chromosome compaction activity [93,94]. This contrasts with the activity of condensin I^DC^, which remains associated with chromosomes throughout the cell cycle. Whether its localization is regulated by phosphorylation is not known. Although there is no evidence for condensin’s involvement in XCI in mammals, the related complex cohesin has been linked to chromosome topology changes in the inactive X [95,96]. Notwithstanding, condensin has been implicated in interphase gene regulation and chromosome territory formation in other contexts, for example [97,98,99].

Even though dosage compensation involves repression in both mammals and *C. elegans,* the structural changes induced on the X chromosomes are very different. In *C. elegans* dosage compensation, the DCC establishes new loop domains to modulate transcription while preserving overall chromosome accessibility. Thus, in *C. elegans*, DCC actively creates chromosome topology across an otherwise active chromosome. In mammals, Xist-driven condensation removes active architecture and builds a facultative heterochromatin compartment that enforces near-complete transcriptional repression. In both cases, chromosome architecture is important in dosage compensation output, but the directionality and molecular mechanisms of architectural change are fundamentally distinct (Figure 4).

In *C. elegans*, genetic evidence suggests that this structural scaffold contributes to the establishment of dosage compensation. In SDC-2-depleted XX hermaphrodites, the TAD boundary formation is lost. In these mutants, the insulation profile of the X chromosome was greatly reduced and was accompanied by elevated X-linked gene expression, suggesting a link between TAD formation and gene repression [91]. However, deletion of the eight strongest rex sites leads to disruption of TAD formation, but only minimal changes in gene expression, implying that these topological changes are not required for gene repression. Hermaphrodite worms with rex site deletions are shorter-lived, which suggests that proper X-chromosome topology promotes longevity even if it is not strictly required for repression [89]. These observations suggest that these topological changes are not the major drivers of gene expression changes in either system.

#### 3.3.2. Chromatin Modifications and Nuclear Architecture

In parallel with large-scale architectural reorganization, establishment of dosage compensation on the *C. elegans* X chromosomes is accompanied by changes in chromatin state. As in mammals, the X-chromosome acquires a unique set and distribution of histone modifications, but the actual modifications are different between the species. Mammals almost completely silence the inactive X, while *C. elegans* only dampens expression, requiring a different set of chromatin marks. While there is a general reduction in active chromatin marks on the dosage compensated for X in *C. elegans,* the degree of depletion does not reach the levels seen on the mammalian inactive X [90,100]. The best characterized modification associated with the *C. elegans* dosage-compensated X is the repressive mark monomethylation of histone H4 at lysine 20 (H4K20me1), a mark also seen on the mammalian inactive X. Immunofluorescence microscopy and genome-wide chromatin profiling demonstrated that H4K20me1 is selectively enriched across X chromosomes in XX *C. elegans* hermaphrodites and that this enrichment depends on a functional Dosage Compensation Complex [100,101,102].

Deposition of H4K20me1 onto unmethylated histone H4 is catalyzed by SET-1, the *C. elegans* ortholog of mammalian SETD8/PR-Set7, whereas conversion of monomethylation to di- and trimethylation of H4K20 (H4K20me3) is mediated by SET-4, the ortholog of Suv4-20 [100,102]. During dosage compensation, the DCC subunit DPY-21, a member of the Jumonji C family of demethylases, converts me2/me3 back to H4K20me1 selectively on the X [101]. H4K20me1 reduction due to *set-1* or *dpy-21* null mutation led to a significant increase in X chromosome expression compared to autosomes in L3 larvae or embryos [30,101,103]. Importantly, DCC localization to the X-chromosome remains intact in *set-1*, *set-4* and *dpy-21* mutants [100,103,104], indicating that H4K20me1 acts downstream of DCC recruitment and contributes specifically to establishment of transcriptional repression rather than to targeting of the complex. H4K20me1 is thought to reinforce the structural changes imposed by condensin I^DC^ [101]. Importantly, DCC begins to repress X chromosomes in early embryogenesis (40- cell stage) before DPY-21-mediated H4K20me1 enrichment on the X happens between 100-cell to comma stage [27,102,103]. This may suggest that condensin I^DC^-mediated loop structures gain enhanced stability when embedded within an H4K20me1-enriched chromatin environment, increasing resistance to transcriptional activation. H4K20me1 is required for mitotic chromosome condensation in mammals, suggesting that H4K20me1 enrichment on the X may reduce the access of transcription machinery to X-linked genes by inducing chromatin compaction [102,105].

Chromatin modification and chromosome architecture are tightly coupled during establishment of dosage compensation. Depletion of H4K20me1 results in weakened repression, reduced chromatin compaction, and impaired domain insulation, despite continued presence of condensin I^DC^ on the X [30,87,101]. DPY-21 regulates the dynamics of condensinI^DC^ binding, which is important for transcription repression [106]. These results suggest that establishment of dosage compensation relies on reciprocal reinforcement between chromosome structure and chromatin state. Condensin I^DC^-mediated topology provides a spatial framework that promotes selective chromatin modification, while DPY-21-mediated H4K20me1 enrichment stabilizes this architecture and enhances transcriptional repression.

In addition to chromosome topology and histone modification, nuclear organizations also support establishment of dosage compensation. CEC-4 is a nuclear tethering protein which binds H3K9-methylated chromatin and anchors heterochromatic regions to the nuclear lamina [107]. CEC-4-mediated tethering and the DCC cooperate to compact the X chromosomes and anchor them to the nuclear lamina [108]. Loss of CEC-4 disrupts X-chromosome condensation and subnuclear localization, but this loss does not affect DCC recruitment or H4K20me1 enrichment on X. This indicates that nuclear tethering acts downstream of DCC binding and independently of DCC localization. Also, loss of CEC-4 function led to a modest but significant derepression of X-linked genes, suggesting that CEC-4-mediated nuclear tethering of X chromosomes stabilizes repression but it is not strictly required. Mutations in *cec-4* lead to X decompaction comparable to DCC mutants, but only minimal gene expression changes [108]. This provides evidence that chromosome compaction and gene repression can be uncoupled.

#### 3.3.3. Transcriptional Dampening via Reduced Pol II Binding

Although condensin I^DC^-mediated architectural remodeling and DPY-21-dependent chromatin modification provides the physical and epigenetic framework for dosage compensation, transcriptional analyses demonstrate that reduction in X-linked gene expression is itself a progressive and dynamic process. The transition from a fully active X chromosome to a dosage-compensated state does not occur instantaneously upon DCC recruitment. Instead, transcriptional dampening unfolds in stages, with gene-specific and region-specific kinetics that reflect the interplay between chromosome topology, chromatin state, and transcriptional regulation.

Genome-wide chromatin immunoprecipiation studies mapping RNA Pol II occupancy provided early insight into how transcription is modulated during dosage compensation by limiting Pol II binding to the X chromosomes [86]. DCC-mediated repression can be detected by RNA-seq in early embryos, shortly after DCC recruitment to the X chromosome [103]. This initial reduction is detectable even before full establishment of chromatin modifications, indicating that architectural changes can influence transcriptional engagement at early stages. GRO-seq experiment tracking nascent RNA transcripts also show that dosage compensation reduces the levels of engaged Pol II across X-linked gene bodies in XX hermaphrodites [109]. As embryogenesis proceeds, Pol II depletion becomes more pronounced, particularly in regions with strong condensin I^DC^ binding. Mid-embryonic stages show separation between X chromosomes and autosomes in Pol II occupancy profiles, with the most robust DCC-bound regions exhibiting the earliest and strongest decreases. Thus, *C. elegans* equalizes X-chromosome-wide gene expression between the sexes by reducing Pol II recruitment to the promoters of X-linked genes in XX [109].

## 4. Maintenance of Dosage Compensation

### 4.1. Maintenance of X-Chromosome-Inactivated State in Mammals

Once established, dosage compensation must be maintained for the lifetime of the organism. In mammals, maintenance of dosage compensation through X-chromosome inactivation (XCI) is mechanistically distinct from its initiation and establishment. It relies on epigenetic memory rather than continuous activity of the initiating factor, XIST or the X inactivation center (XIC). Early experiments analyzing derivatives of the human inactive X chromosome in mouse/human somatic cell hybrids suggested that XIC is not required for maintenance of X-chromosome inactivation in somatic cells [28]. Thus, once dosage compensation is established, the inactive X chromosome can be maintained independently of XIST. Even though the conditional deletion of *Xist* in somatic cells led to the disruption of the localization of macroH2A on the inactive X chromosome, it did not cause global reactivation of X-linked genes [110]. This further reveals that maintenance of XCI in differentiated cells does not rely on XIST. Similarly, Xist-mediated repression in embryonic stem (ES) cells and during early XCI is reversible and requires Xist expression [9]. However, after 48–72 h of differentiation, XCI becomes irreversible and independent of Xist. This further implies that XIST is required for XCI initiation and establishment but is dispensable for maintenance.

In mouse embryonic fibroblasts, loss of Xist in the maintenance phase resulted in limited reactivation of X-linked genes [29]. Inhibition of DNA demethylation or suppression of histone deacetylation further exacerbated gene derepression. This implies that Xist interacts with other epigenetic factors to contribute to gene silencing, even though it is not strictly necessary for XCI maintenance [8]. Although XCI can be maintained without *Xist* in differentiated cells under controlled conditions, long-term loss of *Xist* in vivo destabilizes the inactive X chromosome and leads to pathological consequences, linking XCI maintenance to genome integrity and cancer suppression [111].

Mammals rely on epigenetic inheritance systems that are linked to cell cycles to maintain XCI. Later in the X inactivation process, as silencing becomes Xist-independent, additional mechanisms are recruited to the Xi. CpG islands are methylated on cytosine residues on the Xi [112]. This methylation takes place after silencing has been established [113,114]. The methylation marks are placed by the de novo methyltransferase Dnmt3b, and at least at some loci, the process depends on SmcHD1 [113]. SmcHD1 plays important roles in establishing the higher-order structure of the Xi, as described above, but it was originally described as a protein required for X inactivation maintenance [115].

Once established, DNA methylation can be maintained without the initiating trigger. DNA methylation is copied during the S phase by the maintenance DNA methyltransferase Dnmt1 [113]. These methyltransferases recognize hemimethylated DNA and restore full methylation on newly synthesized strands. This provides a heritable means of preserving transcriptional repression across cell divisions.

In addition, histone modifications associated with repressive chromatin may play important roles in long-term maintenance. In addition to the H3K27me3 mark placed by PRC2 described above, the inactive X is also enriched for H3K9 trimethylation (H3K9me3), placed by Setdb1, in the intergenic regions [116]. SmcHD1 is also necessary for the establishment of these blocks of H3K9me3 enrichment on the inactive X. Lack of SmcHD1 leads to the loss of these H3K9me3 blocks and redistribution of the H3K27me3 mark [117]. Importantly, H3K9 methylation can also be inherited through mitosis and can contribute to the maintenance of silent chromatin states [118]. Similarly, Polycomb-mediated H3K27 trimethylation is maintained when PRC2 recognizes pre-existing H3K27me3 marks and catalyzes methylation of newly incorporated histones during DNA replication and across cell divisions [119,120]. Therefore, establishment of these silencing marks sets up a heritable repressed state. Apf7ip, a protein that coordinates DNA methylation and H3K9 methylation and is known to interact with Mbd1 and Setdb1, is also necessary for maintenance of silencing in differentiated cells [121], thus linking H3K9 methylation and DNA methylation. The nuclear matrix protein CIZ1 localizes to the inactive X in earlier stages but only appears to be required for maintenance [122,123].

Finally, the RNA-binding proteins PTBP1, MATR3, CELF1 and TDP-43 bind to the E-repeat region of Xist [124]. The proteins form a condensate which is nucleated by Xist but that can be maintained in its absence. This transition occurs at the time when silencing becomes Xist-independent. E-repeat mutants can establish silencing, but not maintain it, suggesting that condensate formation by these proteins is yet another mechanism that contributes to maintenance [124]. Together, these studies establish that maintenance of mammalian XCI is epigenetic in nature. Although Xist helps preserve long-term stability and genome integrity in vivo once established, the XCI is maintained by self-propagating DNA and histone modifications and nuclear compartmentalization and does not require ongoing XIST activity.

### 4.2. Maintenance of Dosage-Compensated Repressed State in C. elegans

In contrast to mammals, dosage compensation in *C. elegans* does not transition into a self-sustaining, epigenetically locked state after establishment [30]. Instead, maintenance of X-chromosome repression in *C. elegans* requires the continuous presence of condensin I^DC^, ongoing chromatin modification, and sustained nuclear architecture. By the end of embryogenesis in *C. elegans*, dosage compensation is fully established, and most somatic cells have exited mitosis and have become post-mitotic [125]. From this stage onward, the dosage-compensated state must be maintained to promote proper growth, tissue function, and overall fitness. However, unlike in mammals, maintenance in this case does not involve epigenetic inheritance through mitotic divisions. While the molecular mechanisms governing dosage compensation establishment during embryogenesis have been extensively studied, comparatively, less is known about how dosage compensation is maintained after its establishment. Early genetic studies using cold-sensitive *dpy-27* alleles showed that condensin I^DC^ activity is required for survival during mid-embryogenesis, whereas loss of *dpy-27* function at later stages has a much milder impact on viability [16]. These initial observations suggested that DPY-27 is required for dosage compensation establishment, but perhaps not for maintenance. Recent work by Trombley et al. provides a detailed and systematic analysis of dosage compensation maintenance in *C. elegans.* Depletion of DPY-27 using an auxin-inducible degron (AID) system during embryogenesis resulted in nearly complete embryonic lethality, confirming that condensin I^DC^ is indispensable for the establishment of dosage compensation. In contrast, depletion of DPY-27 during larval or adult stages did not compromise survival, although animals exhibited pronounced developmental defects [30]. However, detailed analysis revealed that these larvae survived not because dosage compensation was maintained in the absence of DPY-27, but rather that they survived even though dosage compensation was not maintained. Loss of DPY-27 was associated with dissociation of condensin I^DC^ from the X chromosomes, decondensation of X-chromosome territories, loss of peripheral nuclear positioning, and loss of H4K20me1 enrichment [30]. These changes were accompanied by a significant increase in X-linked gene expression. These results demonstrate that maintenance of dosage compensation is not a passive epigenetic memory but requires continuous and cooperative action of multiple repressive mechanisms [30]. Thus, continuous condensin I^DC^ activity is required to preserve the repressive architecture of the X chromosome and maintain dosage compensation in post-mitotic cells. These findings also reveal that while dosage compensation establishment is required for embryonic viability, maintenance of dosage compensation in larvae and adults is critical for normal development and for overall fitness, but not viability.

Maintenance of dosage compensation in *C. elegans* also requires the continuous activity of DPY-21, which enriches H4K20me1 on X chromosomes [101]. In post-mitotic cells, H4K20me1 enrichment on X chromosomes is gradually lost in DPY-27-depleted worms, despite having been deposited earlier during embryogenesis [30]. Thus, H4K20me1 is not a long-term epigenetic memory mark and hence requires continuous replenishment, possibly through ongoing recruitment of DPY-21 by condensin I^DC^. The loss of H4K20me1 enrichment is associated with X-chromosome decompaction and transcriptional derepression. This suggests that chromatin modification and chromosome architecture are tightly coupled during maintenance of dosage compensation.

Additionally, the nuclear lamina protein, CEC-4, contributes to maintaining repressed chromatin state. CEC-4 tethers H3K9me3-enriched chromatin to the nuclear periphery and contributes to maintaining the compact and peripheral localization of the dosage-compensated X chromosome [108]. While loss of CEC-4 alone causes only mild defects in X-chromosome repression, loss of CEC-4 function mutation exacerbated X-linked gene derepression [30]. Together, these findings support a model in which maintenance of dosage compensation in *C. elegans* depends on the continuous presence of condensin I^DC^, DPY-21 and CEC- 4. Yet, it is important to note that the defects observed in *cec-4* mutants following DPY-27 depletion cannot be attributed solely to a direct role for CEC- 4 in dosage compensation maintenance, as the CEC- 4 function was lacking at all stages of development. It will therefore be interesting in future to use the AID system to study the function of CEC- 4 exclusively in post-mitotic cells to assess its potential role in establishment versus maintenance of dosage compensation. It is also unknown whether the non-condensin subunits of DCC, especially SDC-2, are required for maintaining the already established dosage-compensated state in post-mitotic cells. It will also be interesting to investigate whether temporal depletion and recovery of DCC might result in re-establishment and maintenance of dosage compensation in post-mitotic cells.

## 5. Conclusions

Studies of dosage compensation enable us to understand how chromosome-wide gene repression is established and maintained in organisms. In this review, we highlight that although both mammals and *Caenorhabditis elegans* achieve dosage compensation through repression of X-linked gene expression, the mechanisms of initiation, establishment, and maintenance are different. These comparisons highlight that different lineages evolved to make use of distinct molecular machinery to solve the same issue: unequal gene expression from the sex chromosomes in the different sexes. In mammals, X-chromosome inactivation is initiated by XIST, and once established, it is maintained through DNA methylation, heritable histone modifications, and nuclear compartmentalization, with XIST contributing primarily to long-term stability and genome integrity. In contrast, *C. elegans* dosage compensation maintenance requires the continuous activity of the initiating machinery itself, including condensin I^DC^, enrichment of H4K20me1, and reinforcement through CEC-4-mediated nuclear lamina tethering. Thus, maintenance of dosage compensation in *C. elegans* is an active, dynamic process rather than passive epigenetic memory. There are still gaps in understanding how dosage compensation is maintained once established in *C. elegans.* This includes whether SDC-2, the protein that triggers DCC assembly on the X, is required for maintenance. Also, the role of CEC-4 in establishment and maintenance has not been decoupled. Future experiments in these areas would enhance our understanding of how the repressed chromatin state is maintained in *C. elegans.*

## Data Availability

No new data were created or analyzed in this study. Data sharing is not applicable to this article.

## Abbreviations

The following abbreviations are used in this manuscript:

XCI	X Chromosome Inactivation
MSL	Male-Specific Lethal
DCC	Dosage Compensation Complex
PRC	Polycomb repressive complex
XIC	X inactivation center
SMC2	Structural Maintenance of Chromosomes 2
CAPG-1	Chromosome-associated polypeptide G
PSC	pluripotent stem cell
ICM	inner cell mass
ESC	embryonic stem cell
TADs	Topologically Associated Domains (TADs)

## References

[1] Koopman P., Gubbay J., Vivian N., Goodfellow P., Lovell-Badge R. (1991). Male development of chromosomally female mice transgenic for Sry. Nature.

[2] Meyer B.J. (2022). Mechanisms of sex determination and X-chromosome dosage compensation. Genetics.

[3] Lyon M.F. (1961). Gene Action in the X-chromosome of the Mouse (*Mus musculus* L.). Nature.

[4] Dror I., Tan T., Plath K. (2024). A critical role for X-chromosome architecture in mammalian X-chromosome dosage compensation. Curr. Opin. Genet. Dev..

[5] Borsani G., Tonlorenzi R., Simmler M.C., Dandolo L., Arnaud D., Capra V., Grompe M., Pizzuti A., Muzny D., Lawrence C. (1991). Characterization of a murine gene expressed from the inactive X chromosome. Nature.

[6] Brown C.J., Ballabio A., Rupert J.L., Lafreniere R.G., Grompe M., Tonlorenzi R., Willard H.F. (1991). A gene from the region of the human X inactivation centre is expressed exclusively from the inactive X chromosome. Nature.

[7] Brockdorff N., Ashworth A., Kay G.F., Cooper P., Smith S., McCabe V.M., Norris D.P., Penny G.D., Patel D., Rastan S. (1991). Conservation of position and exclusive expression of mouse Xist from the inactive X chromosome. Nature.

[8] Bowness J.S., Nesterova T.B., Wei G., Rodermund L., Almeida M., Coker H., Carter E.J., Kadaster A., Brockdorff N. (2022). Xist-mediated silencing requires additive functions of SPEN and Polycomb together with differentiation-dependent recruitment of SmcHD1. Cell Rep..

[9] Wutz A., Jaenisch R. (2000). A Shift from Reversible to Irreversible X Inactivation Is Triggered during ES Cell Differentiation. Mol. Cell.

[10] Donohoe M.E., Silva S.S., Pinter S.F., Xu N., Lee J.T. (2009). The pluripotency factor Oct4 interacts with Ctcf and also controls X-chromosome pairing and counting. Nature.

[11] Navarro P., Festuccia N., Colby D., Gagliardi A., Mullin N.P., Zhang W., Karwacki-Neisius V., Osorno R., Kelly D., Robertson M. (2012). OCT4/SOX2-independent Nanog autorepression modulates heterogeneous Nanog gene expression in mouse ES cells. EMBO J..

[12] Navarro P., Chambers I., Karwacki-Neisius V., Chureau C., Morey C., Rougeulle C., Avner P. (2008). Molecular Coupling of *Xist* Regulation and Pluripotency. Science.

[13] Meyer B.J., Casson L.P. (1986). Caenorhabditis elegans compensates for the difference in X chromosome dosage between the sexes by regulating transcript levels. Cell.

[14] Hodgkin J. (1983). X chromosome dosage and gene expression in Caenorhabditis elegans: Two unusual dumpy genes. Mol. Genet. Genom..

[15] Meyer B.J. (2005). X-Chromosome dosage compensation. WormBook.

[16] Plenefisch J.D., DeLong L., Meyer B.J. (1989). Genes that implement the hermaphrodite mode of dosage compensation in Caenorhabditis elegans. Genetics.

[17] Hirano T. (2005). Condensins: Organizing and Segregating the Genome. Curr. Biol..

[18] Belmont A.S. (2006). Mitotic chromosome structure and condensation. Curr. Opin. Cell Biol..

[19] Ono T., Losada A., Hirano M., Myers M.P., Neuwald A.F., Hirano T. (2003). Differential Contributions of Condensin I and Condensin II to Mitotic Chromosome Architecture in Vertebrate Cells. Cell.

[20] Ono T., Fang Y., Spector D.L., Hirano T. (2004). Spatial and Temporal Regulation of Condensins I and II in Mitotic Chromosome Assembly in Human Cells. Mol. Biol. Cell.

[21] Hirota T., Gerlich D., Koch B., Ellenberg J., Peters J.-M. (2004). Distinct functions of condensin I and II in mitotic chromosome assembly. J. Cell Sci..

[22] Hirano T. (2012). Condensins: Universal organizers of chromosomes with diverse functions. Genes Dev..

[23] Csankovszki G., Collette K., Spahl K., Carey J., Snyder M., Petty E., Patel U., Tabuchi T., Liu H., McLeod I. (2009). Three Distinct Condensin Complexes Control C. elegans Chromosome Dynamics. Curr. Biol..

[24] Chawla B., Csankovszki G. (2024). How Chromatin Motor Complexes Influence the Nuclear Architecture: A Review of Chromatin Organization, Cohesins, and Condensins with a Focus on *C. elegans*. DNA.

[25] Chuang P.-T., Albertson D.G., Meyer B.J. (1994). DPY-27: A chromosome condensation protein homolog that regulates C. elegans dosage compensation through association with the X chromosome. Cell.

[26] Albritton S.E., Ercan S. (2018). Caenorhabditis elegans Dosage Compensation: Insights into Condensin-Mediated Gene Regulation. Trends Genet..

[27] Custer L.M., Snyder M.J., Flegel K., Csankovszki G. (2014). The onset of C. elegans dosage compensation is linked to the loss of developmental plasticity. Dev. Biol..

[28] Brown C.J., Willard H.F. (1994). The human X-inactivation centre is not required for maintenance of X-chromosome inactivation. Nature.

[29] Csankovszki G., Nagy A., Jaenisch R. (2001). Synergism of Xist Rna, DNA Methylation, and Histone Hypoacetylation in Maintaining X Chromosome Inactivation. J. Cell Biol..

[30] Trombley J., Rakozy A.I., McClear C.A., Jash E., Csankovszki G. (2025). Condensin IDC, DPY-21, and CEC-4 maintain X chromosome repression in C. elegans. PLoS Genet..

[31] Du Z., Hu L., Zou Z., Liu M., Li Z., Lu X., Harris C., Xiang Y., Chen F., Yu G. (2024). Stepwise de novo establishment of inactive X chromosome architecture in early development. Nat. Genet..

[32] Berg I.M.v.D., Galjaard R.J., Laven J.S.E., van Doorninck J.H. (2011). XCI in preimplantation mouse and human embryos: First there is remodelling…. Hum. Genet..

[33] Loda A., Collombet S., Heard E. (2022). Gene regulation in time and space during X-chromosome inactivation. Nat. Rev. Mol. Cell Biol..

[34] Okamoto I., Otte A.P., Allis C.D., Reinberg D., Heard E. (2004). Epigenetic Dynamics of Imprinted X Inactivation During Early Mouse Development. Science.

[35] Huynh K.D., Lee J.T. (2003). Inheritance of a pre-inactivated paternal X chromosome in early mouse embryos. Nature.

[36] Takagi N., Sasaki M. (1975). Preferential inactivation of the paternally derived X chromosome in the extraembryonic membranes of the mouse. Nature.

[37] Payer B., Lee J.T. (2008). X Chromosome Dosage Compensation: How Mammals Keep the Balance. Annu. Rev. Genet..

[38] Sahakyan A., Kim R., Chronis C., Sabri S., Bonora G., Theunissen T.W., Kuoy E., Langerman J., Clark A.T., Jaenisch R. (2017). Human Naive Pluripotent Stem Cells Model X Chromosome Dampening and X Inactivation. Cell Stem Cell.

[39] Petropoulos S., Edsgärd D., Reinius B., Deng Q., Panula S.P., Codeluppi S., Reyes A.P., Linnarsson S., Sandberg R., Lanner F. (2016). Single-Cell RNA-Seq Reveals Lineage and X Chromosome Dynamics in Human Preimplantation Embryos. Cell.

[40] Okamoto I., Patrat C., Thépot D., Peynot N., Fauque P., Daniel N., Diabangouaya P., Wolf J.-P., Renard J.-P., Duranthon V. (2011). Eutherian mammals use diverse strategies to initiate X-chromosome inactivation during development. Nature.

[41] Alfeghaly C., Castel G., Cazottes E., Moscatelli M., Moinard E., Casanova M., Boni J., Mahadik K., Lammers J., Freour T. (2024). XIST dampens X chromosome activity in a SPEN-dependent manner during early human development. Nat. Struct. Mol. Biol..

[42] Vallot C., Patrat C., Collier A.J., Huret C., Casanova M., Ali T.M.L., Tosolini M., Frydman N., Heard E., Rugg-Gunn P.J. (2017). XACT Noncoding RNA Competes with XIST in the Control of X Chromosome Activity during Human Early Development. Cell Stem Cell.

[43] Masui S., Nakatake Y., Toyooka Y., Shimosato D., Yagi R., Takahashi K., Okochi H., Okuda A., Matoba R., Sharov A.A. (2007). Pluripotency governed by Sox2 via regulation of Oct3/4 expression in mouse embryonic stem cells. Nat. Cell Biol..

[44] Mitsui K., Tokuzawa Y., Itoh H., Segawa K., Murakami M., Takahashi K., Maruyama M., Maeda M., Yamanaka S. (2003). The Homeoprotein Nanog Is Required for Maintenance of Pluripotency in Mouse Epiblast and ES Cells. Cell.

[45] Nichols J., Zevnik B., Anastassiadis K., Niwa H., Klewe-Nebenius D., Chambers I., Schöler H., Smith A. (1998). Formation of Pluripotent Stem Cells in the Mammalian Embryo Depends on the POU Transcription Factor Oct4. Cell.

[46] Nesterova T.B., Wei G., Coker H., Pintacuda G., Bowness J.S., Zhang T., Almeida M., Bloechl B., Moindrot B., Carter E.J. (2019). Systematic allelic analysis defines the interplay of key pathways in X chromosome inactivation. Nat. Commun..

[47] Donohoe M.E., Zhang L.-F., Xu N., Shi Y., Lee J.T. (2007). Identification of a Ctcf Cofactor, Yy1, for the X Chromosome Binary Switch. Mol. Cell.

[48] Ogawa Y., Lee J.T. (2003). Xite, X-Inactivation Intergenic Transcription Elements that Regulate the Probability of Choice. Mol. Cell.

[49] Lee J.T., Lu N. (1999). Targeted Mutagenesis of Tsix Leads to Nonrandom X Inactivation. Cell.

[50] Richly H., Aloia L., Di Croce L. (2011). Roles of the Polycomb group proteins in stem cells and cancer. Cell Death Dis..

[51] Zepeda-Martinez J.A., Pribitzer C., Wang J., Bsteh D., Golumbeanu S., Zhao Q., Burkard T.R., Reichholf B., Rhie S.K., Jude J. (2020). Parallel PRC2/cPRC1 and vPRC1 pathways silence lineage-specific genes and maintain self-renewal in mouse embryonic stem cells. Sci. Adv..

[52] Engreitz J.M., Pandya-Jones A., McDonel P., Shishkin A., Sirokman K., Surka C., Kadri S., Xing J., Goren A., Lander E.S. (2013). The Xist lncRNA Exploits Three-Dimensional Genome Architecture to Spread Across the X Chromosome. Science.

[53] Monfort A., Di Minin G., Postlmayr A., Freimann R., Arieti F., Thore S., Wutz A. (2015). Identification of Spen as a Crucial Factor for Xist Function through Forward Genetic Screening in Haploid Embryonic Stem Cells. Cell Rep..

[54] Moindrot B., Cerase A., Coker H., Masui O., Grijzenhout A., Pintacuda G., Schermelleh L., Nesterova T.B., Brockdorff N. (2015). A Pooled shRNA Screen Identifies Rbm15, Spen, and Wtap as Factors Required for Xist RNA-Mediated Silencing. Cell Rep..

[55] Dossin F., Pinheiro I., Zylicz J., Roensch J., Collombet S., Le Saux A., Chelmicki T., Attia M., Kapoor V., Zhan Y. (2020). SPEN integrates transcriptional and epigenetic control of X-inactivation. Nature.

[56] Chu C., Zhang Q.C., da Rocha S.T., Flynn R.A., Bharadwaj M., Calabrese J.M., Magnuson T., Heard E., Chang H.Y. (2015). Systematic Discovery of Xist RNA Binding Proteins. Cell.

[57] McHugh C.A., Chen C.-K., Chow A., Surka C.F., Tran C., McDonel P., Pandya-Jones A., Blanco M., Burghard C., Moradian A. (2015). The Xist lncRNA interacts directly with SHARP to silence transcription through HDAC3. Nature.

[58] Wang C.-Y., Colognori D., Sunwoo H., Wang D., Lee J.T. (2019). PRC1 collaborates with SMCHD1 to fold the X-chromosome and spread Xist RNA between chromosome compartments. Nat. Commun..

[59] Pintacuda G., Wei G., Roustan C., Kirmizitas B.A., Solcan N., Cerase A., Castello A., Mohammed S., Moindrot B., Nesterova T.B. (2017). hnRNPK Recruits PCGF3/5-PRC1 to the Xist RNA B-Repeat to Establish Polycomb-Mediated Chromosomal Silencing. Mol. Cell.

[60] JanszN, NesterovaT, KeniryA, IminitoffM, HickeyP, PintacudaG, MasuiO, KobelkeS, GeogheganN, BreslinK A. (2018). Smchd1 Targeting to the Inactive X Is Dependent on the Xist-HnrnpK-PRC1 Pathway. Cell Rep..

[61] Colognori D., Sunwoo H., Kriz A.J., Wang C.-Y., Lee J.T. (2019). Xist Deletional Analysis Reveals an Interdependency between Xist RNA and Polycomb Complexes for Spreading along the Inactive X. Mol. Cell.

[62] Bousard A., Raposo A.C., Żylicz J.J., Picard C., Pires V.B., Qi Y., Gil C., Syx L., Chang H.Y., Heard E. (2019). The role of *Xist* -mediated Polycomb recruitment in the initiation of X-chromosome inactivation. Embo Rep..

[63] Almeida M., Pintacuda G., Masui O., Koseki Y., Gdula M., Cerase A., Brown D., Mould A., Innocent C., Nakayama M. (2017). PCGF3/5–PRC1 initiates Polycomb recruitment in X chromosome inactivation. Science.

[64] Tjalsma S.J.D., Hori M., Sato Y., Bousard A., Ohi A., Raposo A.C., Roensch J., Le Saux A., Nogami J., Maehara K. (2021). H4K20me1 and H3K27me3 are concurrently loaded onto the inactive X chromosome but dispensable for inducing gene silencing. Embo Rep..

[65] Kohlmaier A., Savarese F., Lachner M., Martens J., Jenuwein T., Wutz A. (2004). A Chromosomal Memory Triggered by Xist Regulates Histone Methylation in X Inactivation. PLOS Biol..

[66] Wang C.-Y., Jégu T., Chu H.-P., Oh H.J., Lee J.T. (2018). SMCHD1 Merges Chromosome Compartments and Assists Formation of Super-Structures on the Inactive X. Cell.

[67] Rodermund L., Coker H., Oldenkamp R., Wei G., Bowness J., Rajkumar B., Nesterova T., Pinto D.M.S., Schermelleh L., Brockdorff N. (2021). Time-resolved structured illumination microscopy reveals key principles of Xist RNA spreading. Science.

[68] Markaki Y., Chong J.G., Wang Y., Jacobson E.C., Luong C., Tan S.Y., Jachowicz J.W., Strehle M., Maestrini D., Banerjee A.K. (2021). Xist nucleates local protein gradients to propagate silencing across the X chromosome. Cell.

[69] Giorgetti L., Lajoie B.R., Carter A.C., Attia M., Zhan Y., Xu J., Chen C.J., Kaplan N., Chang H.Y., Heard E. (2016). Structural organization of the inactive X chromosome in the mouse. Nature.

[70] Rao S.S.P., Huntley M.H., Durand N.C., Stamenova E.K., Bochkov I.D., Robinson J.T., Sanborn A.L., Machol I., Omer A.D., Lander E.S. (2014). A 3D Map of the Human Genome at Kilobase Resolution Reveals Principles of Chromatin Looping. Cell.

[71] Deng X., Ma W., Ramani V., Hill A., Yang F., Ay F., Berletch J.B., Blau C.A., Shendure J., Duan Z. (2015). Bipartite structure of the inactive mouse X chromosome. Genome Biol..

[72] Darrow E.M., Huntley M.H., Dudchenko O., Stamenova E.K., Durand N.C., Sun Z., Huang S.-C., Sanborn A.L., Machol I., Shamim M. (2016). Deletion of *DXZ4* on the human inactive X chromosome alters higher-order genome architecture. Proc. Natl. Acad. Sci. USA.

[73] Froberg J.E., Pinter S.F., Kriz A.J., Jégu T., Lee J.T. (2018). Megadomains and superloops form dynamically but are dispensable for X-chromosome inactivation and gene escape. Nat. Commun..

[74] Yuzyuk T., Fakhouri T., Kiefer J., Mango S. (2009). The Polycomb Complex Protein mes-2/E(z) Promotes the Transition from Developmental Plasticity to Differentiation in C. elegans Embryos. Dev. Cell.

[75] Kimble J., Seidel H. (2013). C. elegans Germline Stem Cells and Their Niche.

[76] Joshi P.M., Riddle M.R., Djabrayan N.J., Rothman J.H. (2010). *Caenorhabditis elegans* as a model for stem cell biology. Dev. Dyn..

[77] Dawes H.E., Berlin D.S., Lapidus D.M., Nusbaum C., Davis T.L., Meyer B.J. (1999). Dosage Compensation Proteins Targeted to X Chromosomes by a Determinant of Hermaphrodite Fate. Science.

[78] Miller L.M., Plenefisch J.D., Casson L.P., Meyer B.J. (1988). xol-1: A gene that controls the male modes of both sex determination and X chromosome dosage compensation in C. elegans. Cell.

[79] Rhind N.R., Miller L.M., Kopczynski J.B., Meyer B.J. (1995). xo1-1 acts as an early switch in the C. elegans male/hermaphrodite decision. Cell.

[80] Hargitai B., Kutnyánszky V., Blauwkamp T.A., Steták A., Csankovszki G., Takács-Vellai K., Vellai T. (2009). *xol-1*, the master sex-switch gene in *C. elegans*, is a transcriptional target of the terminal sex-determining factor TRA-1. Development.

[81] Strome S., Kelly W.G., Ercan S., Lieb J.D. (2014). Regulation of the X Chromosomes in Caenorhabditis elegans. Cold Spring Harb. Perspect. Biol..

[82] Csankovszki G., McDonel P., Meyer B.J. (2004). Recruitment and Spreading of the *C. elegans* Dosage Compensation Complex Along X Chromosomes. Science.

[83] McDonel P., Jans J., Peterson B.K., Meyer B.J. (2006). Clustered DNA motifs mark X chromosomes for repression by a dosage compensation complex. Nature.

[84] Jans J., Gladden J.M., Ralston E.J., Pickle C.S., Michel A.H., Pferdehirt R.R., Eisen M.B., Meyer B.J. (2009). A condensin-like dosage compensation complex acts at a distance to control expression throughout the genome. Genes Dev..

[85] Ercan S., Giresi P.G., Whittle C.M., Zhang X., Green R.D., Lieb J.D. (2007). X chromosome repression by localization of the C. elegans dosage compensation machinery to sites of transcription initiation. Nat. Genet..

[86] Pferdehirt R.R., Kruesi W.S., Meyer B.J. (2011). An MLL/COMPASS subunit functions in the *C. elegans* dosage compensation complex to target X chromosomes for transcriptional regulation of gene expression. Genes Dev..

[87] Lau A.C., Nabeshima K., Csankovszki G. (2014). The *C. elegans* dosage compensation complex mediates interphase X chromosome compaction. Epigenetics Chromatin.

[88] Kim J., Jimenez D.S., Ragipani B., Zhang B., A Street L., Kramer M., E Albritton S., Winterkorn L.H., Morao A.K., Ercan S. (2022). Condensin DC loads and spreads from recruitment sites to create loop-anchored TADs in C. elegans. eLife.

[89] Anderson E.C., Frankino P.A., Higuchi-Sanabria R., Yang Q., Bian Q., Podshivalova K., Shin A., Kenyon C., Dillin A., Meyer B.J. (2019). X Chromosome Domain Architecture Regulates Caenorhabditis elegans Lifespan but Not Dosage Compensation. Dev. Cell.

[90] Street L.A., Morao A.K., Winterkorn L.H., Jiao C.-Y., Albritton S.E., Sadic M., Kramer M., Ercan S. (2019). Binding of an *X*-Specific Condensin Correlates with a Reduction in Active Histone Modifications at Gene Regulatory Elements. Genetics.

[91] Crane E., Bian Q., McCord R.P., Lajoie B.R., Wheeler B.S., Ralston E.J., Uzawa S., Dekker J., Meyer B.J. (2015). Condensin-driven remodelling of X chromosome topology during dosage compensation. Nature.

[92] Rowley M.J., Poulet A., Nichols M.H., Bixler B.J., Sanborn A.L., Brouhard E.A., Hermetz K., Linsenbaum H., Csankovszki G., Aiden E.L. (2020). Analysis of Hi-C data using SIP effectively identifies loops in organisms from *C. elegans* to mammals. Genome Res..

[93] Borsellini A., Conti D., Cutts E.E., Harris R.J., Walstein K., Graziadei A., Cecatiello V., Aarts T.F., Xie R., Mazouzi A. (2025). Condensin II activation by M18BP1. Mol. Cell.

[94] Borsellini A. (2026). A phospho-switch to trigger mitotic chromosome condensation. Biol. Direct.

[95] Generoso S.F., Neguembor M.V., Hershberg E.A., Sadreyev R.I., Kurimoto K., Yabuta Y., Ricci R., Audergon P., Bauer M., Saitou M. (2023). Cohesin controls X chromosome structure remodeling and X-reactivation during mouse iPSC-reprogramming. Proc. Natl. Acad. Sci. USA.

[96] Kriz A.J., Colognori D., Sunwoo H., Nabet B., Lee J.T. (2021). Balancing cohesin eviction and retention prevents aberrant chromosomal interactions, Polycomb-mediated repression, and X-inactivation. Mol. Cell.

[97] Iwasaki O., Tanizawa H., Kim K.-D., Kossenkov A., Nacarelli T., Tashiro S., Majumdar S., Showe L.C., Zhang R., Noma K.-I. (2019). Involvement of condensin in cellular senescence through gene regulation and compartmental reorganization. Nat. Commun..

[98] Bauer C.R., Hartl T.A., Bosco G. (2012). Condensin II Promotes the Formation of Chromosome Territories by Inducing Axial Compaction of Polyploid Interphase Chromosomes. PLoS Genet..

[99] Hassan A., Rodriguez P.A., Heidmann S.K., Walmsley E.L., Aughey G.N., Southall T.D. (2020). Condensin I subunit Cap-G is essential for proper gene expression during the maturation of post-mitotic neurons. eLife.

[100] Wells M.B., Snyder M.J., Custer L.M., Csankovszki G. (2012). *Caenorhabditis elegans* Dosage Compensation Regulates Histone H4 Chromatin State on X Chromosomes. Mol. Cell. Biol..

[101] Brejc K., Bian Q., Uzawa S., Wheeler B.S., Anderson E.C., King D.S., Kranzusch P.J., Preston C.G., Meyer B.J. (2017). Dynamic Control of X Chromosome Conformation and Repression by a Histone H4K20 Demethylase. Cell.

[102] Vielle A., Lang J., Dong Y., Ercan S., Kotwaliwale C., Rechtsteiner A., Appert A., Chen Q.B., Dose A., Egelhofer T. (2012). H4K20me1 Contributes to Downregulation of X-Linked Genes for C. elegans Dosage Compensation. PLoS Genet..

[103] Kramer M., Kranz A.-L., Su A., Winterkorn L.H., Albritton S.E., Ercan S. (2015). Developmental Dynamics of X-Chromosome Dosage Compensation by the DCC and H4K20me1 in C. elegans. PLoS Genet..

[104] Yonker S.A., Meyer B.J. (2003). Recruitment of *C. elegans* dosage compensation proteins for gene-specific versus chromosome-wide repression. Development.

[105] Oda H., Okamoto I., Murphy N., Chu J., Price S.M., Shen M.M., Torres-Padilla M.E., Heard E., Reinberg D. (2009). Monomethylation of Histone H4-Lysine 20 Is Involved in Chromosome Structure and Stability and Is Essential for Mouse Development. Mol. Cell. Biol..

[106] Breimann L., Morao A.K., Kim J., Jimenez D.S., Maryn N., Bikkasani K., Carrozza M.J., Albritton S.E., Kramer M., Street L.A. (2022). The histone H4 lysine 20 demethylase DPY-21 regulates the dynamics of condensin DC binding. J. Cell Sci..

[107] Gonzalez-Sandoval A., Towbin B.D., Kalck V., Cabianca D.S., Gaidatzis D., Hauer M.H., Geng L., Wang L., Yang T., Wang X. (2015). Perinuclear Anchoring of H3K9-Methylated Chromatin Stabilizes Induced Cell Fate in C. elegans Embryos. Cell.

[108] Snyder M.J., Lau A.C., Brouhard E.A., Davis M.B., Jiang J., Sifuentes M.H., Csankovszki G. (2016). Anchoring of Heterochromatin to the Nuclear Lamina Reinforces Dosage Compensation-Mediated Gene Repression. PLoS Genet..

[109] Kruesi W.S., Core L.J., Waters C.T., Lis J.T., Meyer B.J. (2013). Condensin controls recruitment of RNA polymerase II to achieve nematode X-chromosome dosage compensation. eLife.

[110] Csankovszki G., Panning B., Bates B., Pehrson J.R., Jaenisch R. (1999). Conditional deletion of Xist disrupts histone macroH2A localization but not maintenance of X inactivation. Nat. Genet..

[111] Yildirim E., Kirby J.E., Brown D.E., Mercier F.E., Sadreyev R.I., Scadden D.T., Lee J.T. (2013). Xist RNA Is a Potent Suppressor of Hematologic Cancer in Mice. Cell.

[112] Norris D.P., Brockdorff N., Rastan S. (1991). Methylation status of CpG-rich islands on active and inactive mouse X chromosomes. Mamm. Genome.

[113] Gendrel A.-V., Apedaile A., Coker H., Termanis A., Zvetkova I., Godwin J., Tang Y.A., Huntley D., Montana G., Taylor S. (2012). Smchd1-Dependent and -Independent Pathways Determine Developmental Dynamics of CpG Island Methylation on the Inactive X Chromosome. Dev. Cell.

[114] Lock L.F., Takagi N., Martin G.R. (1987). Methylation of the Hprt gene on the inactive X occurs after chromosome inactivation. Cell.

[115] E Blewitt M., Gendrel A.-V., Pang Z., Sparrow D.B., Whitelaw N., Craig J.M., Apedaile A., Hilton D.J., Dunwoodie S.L., Brockdorff N. (2008). SmcHD1, containing a structural-maintenance-of-chromosomes hinge domain, has a critical role in X inactivation. Nat. Genet..

[116] Keniry A., Gearing L.J., Jansz N., Liu J., Holik A.Z., Hickey P.F., Kinkel S.A., Moore D.L., Breslin K., Chen K. (2016). Setdb1-mediated H3K9 methylation is enriched on the inactive X and plays a role in its epigenetic silencing. Epigenetics Chromatin.

[117] Ichihara S., Nagao K., Sakaguchi T., Obuse C., Sado T. (2022). SmcHD1 underlies the formation of H3K9me3 blocks on the inactive X chromosome in mice. Development.

[118] Audergon P.N.C.B., Catania S., Kagansky A., Tong P., Shukla M., Pidoux A.L., Allshire R.C. (2015). Restricted epigenetic inheritance of H3K9 methylation. Science.

[119] Margueron R., Justin N., Ohno K., Sharpe M.L., Son J., Iii W.J.D., Voigt P., Martin S.R., Taylor W.R., De Marco V. (2009). Role of the polycomb protein EED in the propagation of repressive histone marks. Nature.

[120] Hansen K.H., Bracken A.P., Pasini D., Dietrich N., Gehani S.S., Monrad A., Rappsilber J., Lerdrup M., Helin K. (2008). A model for transmission of the H3K27me3 epigenetic mark. Nat. Cell Biol..

[121] Minkovsky A., Sahakyan A., Rankin-Gee E., Bonora G., Patel S., Plath K. (2014). The Mbd1-Atf7ip-Setdb1 pathway contributes to the maintenance of X chromosome inactivation. Epigenetics Chromatin.

[122] Stewart E.R., Turner R.M.L., Newling K., Ridings-Figueroa R., Scott V., Ashton P.D., Ainscough J.F.X., Coverley D. (2019). Maintenance of epigenetic landscape requires CIZ1 and is corrupted in differentiated fibroblasts in long-term culture. Nat. Commun..

[123] Ridings-Figueroa R., Stewart E.R., Nesterova T.B., Coker H., Pintacuda G., Godwin J., Wilson R., Haslam A., Lilley F., Ruigrok R. (2017). The nuclear matrix protein CIZ1 facilitates localization of Xist RNA to the inactive X-chromosome territory. Genes Dev..

[124] Pandya-Jones A., Markaki Y., Serizay J., Chitiashvili T., Leon W.R.M., Damianov A., Chronis C., Papp B., Chen C.-K., McKee R. (2020). A protein assembly mediates Xist localization and gene silencing. Nature.

[125] Sulston J.E., Schierenberg E., White J.G., Thomson J.N. (1983). The embryonic cell lineage of the nematode Caenorhabditis elegans. Dev. Biol..

